# Bacterial Antigens Reduced the Inhibition Effect of Capsaicin on Cal 27 Oral Cancer Cell Proliferation

**DOI:** 10.3390/ijms22168686

**Published:** 2021-08-12

**Authors:** Rajdeep Chakraborty, Karen Vickery, Charbel Darido, Shoba Ranganathan, Honghua Hu

**Affiliations:** 1 Faculty of Medicine, Health and Human Sciences, Macquarie University, Sydney, NSW 2109, Australia; rajdeep.chakraborty@hdr.mq.edu.au (R.C.); karen.vickery@mq.edu.au (K.V.); 2 Applied Biosciences, Faculty of Science and Engineering, Macquarie University, Sydney, NSW 2109, Australia; shoba.ranganathan@mq.edu.au; 3 Peter MacCallum Cancer Centre, Melbourne, VIC 3000, Australia; charbel.darido@petermac.org; 4 Sir Peter MacCallum Department of Oncology, The University of Melbourne, Melbourne, VIC 3000, Australia

**Keywords:** lipopolysaccharide, lipoteichoic acid, oral cancer, capsaicin, proliferation, apoptosis, proliferation factors, suppressor of cytokine signalling 3

## Abstract

Oral cancer is a major global health problem with high incidence and low survival rates. The oral cavity contains biofilms as dental plaques that harbour both Gram-negative and Gram-positive bacterial antigens, lipopolysaccharide (LPS) and lipoteichoic acid (LTA), respectively. LPS and LTA are known to stimulate cancer cell growth, and the bioactive phytochemical capsaicin has been reported to reverse this effect. Here, we tested the efficacy of oral cancer chemotherapy treatment with capsaicin in the presence of LPS, LTA or the combination of both antigens. LPS and LTA were administered to Cal 27 oral cancer cells prior to and/or concurrently with capsaicin, and the treatment efficacy was evaluated by measuring cell proliferation and apoptotic cell death. We found that while capsaicin inhibits oral cancer cell proliferation and metabolism (MT Glo assay) and increases cell death (Trypan blue exclusion assay and Caspase 3/7 expression), its anti-cancer effect was significantly reduced on cells that are either primed or exposed to the bacterial antigens. Capsaicin treatment significantly increased oral cancer cells’ suppressor of cytokine signalling 3 gene expression. This increase was reversed in the presence of bacterial antigens during treatment. Our data establish a rationale for clinical consideration of bacterial antigens that may interfere with the treatment efficacy of oral cancer.

## 1. Introduction

Oral squamous cell carcinoma (OSCC) is a major global health problem with high incidence and low survival rates. Chronic bacterial infections have been shown to be associated with chronic inflammation, anti-apoptotic activity and pro-proliferative activities, and the Gram-negative, anaerobic bacteria, *Porphyromonas gingivalis* and *Fusobacterium nucleatum* contribute to oral carcinogenesis and progression of oral cancer [1].

Early oral colonisers include Gram-positive bacterial species from the Streptococcus and Actinomyces families which produce lipoteichoic acid (LTA). Late oral colonisers include Gram-negative bacterial species *F. nucleatum*, *P. gingivalis* and *Prevotella* spp., which produce lipopolysaccharide (LPS). Both Gram-positive and Gram-negative bacteria form oral biofilms. Bacterial infection has been shown to be associated with chronic inflammation, anti-apoptotic activity, pro-proliferative activities, which play a role in cancer development [2].

LPS is found in the cell wall of Gram-negative bacteria. Previous studies have shown that *P. gingivalis* LPS tend to increase the viability of oral cancer cells [3]. Studies of the tumour microenvironment have shown that macrophages demonstrate increased IL-6 and CD14 expression and increased nitric oxide secretion after the stimulation of LPS [2]. In macrophages, LPS upregulates tumour necrosis factor alpha (TNFα), that induces suppression of cytokine signalling 3 (*SOCS3*) mRNA expression and inhibits IL-6-induced activation of signal transducer and activator of transcription 3 (*STAT3*) [4]. In contrast, the LPS–squamous cell carcinoma–monocyte interaction leads to rapid STAT3 protein activation and cancer progression [5]. 

LTA from *Staphylococcus aureus* has been shown to stimulate proliferation of human non-small cell lung cancer cells [6]. LTA from *Streptococcus mutans*, *Streptococcus pyogenes*, *Streptococcus faecalis*, *Streptococcus sanguis* and *S. aureus* upregulate Human hepatocyte growth factor (HuHGF) [7], which has been shown to be associated with the progression of oral cancer by stimulating stromal fibroblast-induced invasion (epithelial mesenchymal cells) [8].

Recently, we demonstrated that combined stimulation of oral cancer cells Cal 27 by LPS and LTA resulted in greater proliferation of cells than stimulation with either LPS or LTA alone [9]. 

Capsaicin induces apoptosis in gastric cancer cells, various types of breast cancer as well as in pancreatic, oesophageal, colonic, prostrate, skin, endothelial cell and lung cancer [10]. Capsaicin is reported to reduce the proliferation of an oral squamous cancer cell line of Asian origin, ORL-48 [11], and induces apoptosis in SCC4 and SCC25 oral cancer cells [12].

In the clinical settings, the oral cavity contains oral biofilms as dental plaques that harbour both Gram-negative and Gram-positive bacteria. These bacteria release antigens, which may present prior to and/or during oral cancer chemotherapies given the presence of dental plaque in cancer patients. The aim of this study is to investigate whether the bacterial antigens in the oral cavity affect the efficacy of chemotherapy treatment of oral cancer cells by measuring cell metabolism, viability, and apoptotic cell death. We also investigated the effect of capsaicin on the expression of oral cancer cell proliferation factors SOCS3, EGFR (epidermal growth factor receptor), STAT3 and PI3K (phosphoinositide 3-kinase) in the presence or absence of bacterial antigens.

**Figure 1:**
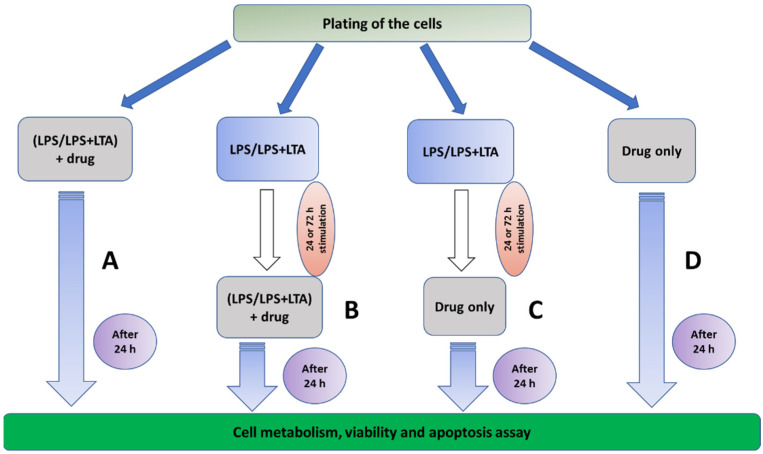
Clinical rationale of bacterial antigens and chemotherapeutic drug combination tests. (**A**) Plated cells were treated with different combinations of bacterial antigens and chemotherapeutic drugs (this group defines the action of chemotherapeutic drugs in the presence of bacterial antigens). (**B**) Inhibitory drugs were administered in the presence of bacterial antigens after 24 or 72 h of bacterial antigen stimulation (these groups define the effect of chemotherapeutic drugs in the presence of bacterial antigens on existing bacterial antigen-stimulated cancer cells, i.e., in patients with existing oral biofilms). (**C**) Inhibitory drugs alone were administered after 24 or 72 h of bacterial antigen stimulation (this group defines the effect of chemotherapeutic drugs on existing bacterial antigen-stimulated cancer cells following clinical removal of biofilm with covering antibiotic therapy). (**D**) Inhibitory drugs were applied in the absence of and without stimulation with bacterial antigens (this group defines a situation where chemotherapeutic drugs are administered to patients who developed oral cancer in the absence of oral biofilm, which is practically impossible in a real-world scenario).

The various capsaicin and LPS/LPS + LTA combination tests represent various clinical scenarios, such as the presence or absence of dental plaque containing Gram-negative and Gram-positive bacterial biofilms providing antigenic stimulation, which is demonstrated in Figure 1.

## 2. Results

### 2.1. Determine the Drug Treatment Time and Titration of Capsaicin

**Figure 2:**
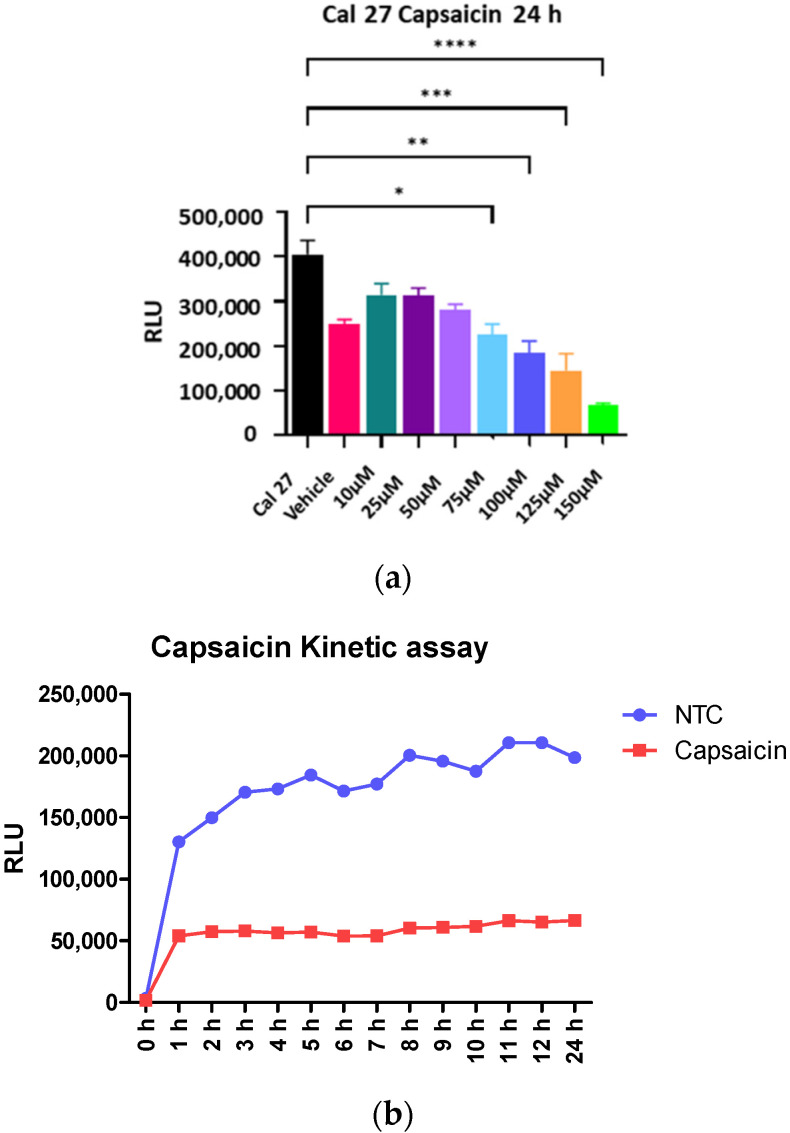
Capsaicin titration. (**a**) Metabolic activity of Cal 27 cells when treated with capsaicin for 24 h. Vehicle is 0.1% DMSO used to solubilise capsaicin. *n* = 6 biological replicates. Error bars represent standard error of the mean. * *p* < 0.05, ** *p* < 0.01, *** *p* < 0.001, **** *p* < 0.0001. (**b**) Effect of 150 µM capsaicin treatment on Cal 27 cells’ metabolism over a 24 h period. The MT Glo kinetic assay was used to measure the metabolic activity of untreated (NTC) and capsaicin-treated Cal 27 over a 24 h period. RLU: relative luminescence unit.

**Figure 3:**
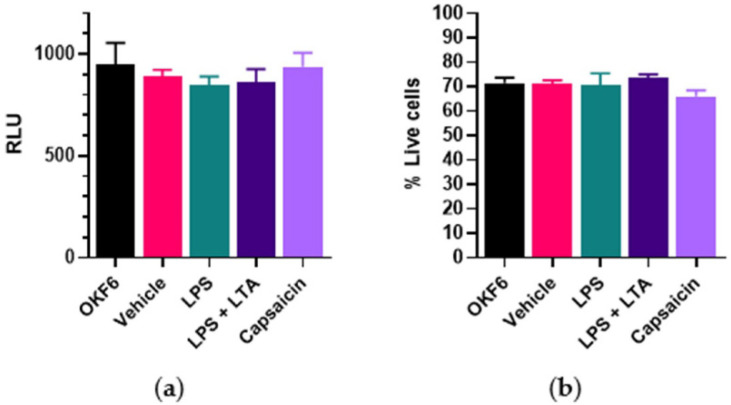
Bacterial antigens and capsaicin treatment on normal oral cell OKF6 metabolism and viability. OKF6 cells were plated at 5000 cells/well and incubated with drug vehicle (0.1% DMSO), LPS (5 µg/mL), combined LPS (5 µg/mL) + LTA (5 µg/mL) and capsaicin (150 µM) for 24 h. (**a**) Cell metabolism was determined by the MT Glo assay. RLU = relative luminescence unit. (**b**) Cell viability was determined by the Trypan blue exclusion assay. *n* = 6 biological replicates. Error bars represent standard error of the mean.

Titration of capsaicin found that the maximum clinically relevant concentration that could be used without adversely affecting normal cells was 150 µM. Capsaicin at 150 µM produced maximum inhibition of oral cancer cell Cal 27 (Figure 2a), but a small effect on cell viability of normal oral cell OKF6 (Figure 3b). Therefore, we could not increase the concentration of capsaicin in the study. Within one hour of application, capsaicin at 150 µM reduced 60% of Cal 27 metabolism (Figure 2b). The level of inhibition remained steady for up to 24 h (Figure 2b).

### 2.2. Effect of Capsaicin Treatment on Normal Oral Cell OKF6 Metabolism and Viability

There was no significant change in normal oral cell OKF6 metabolism (Figure 3a) and a small effect on cell viability (Figure 3b) when treated with 150 µM capsaicin or stimulated with bacterial antigens.

### 2.3. Effect of Capsaicin Treatment on Oral Cancer Cell Metabolism and Viability in the Presence or Absence of Bacterial Antigens

**Figure 4:**
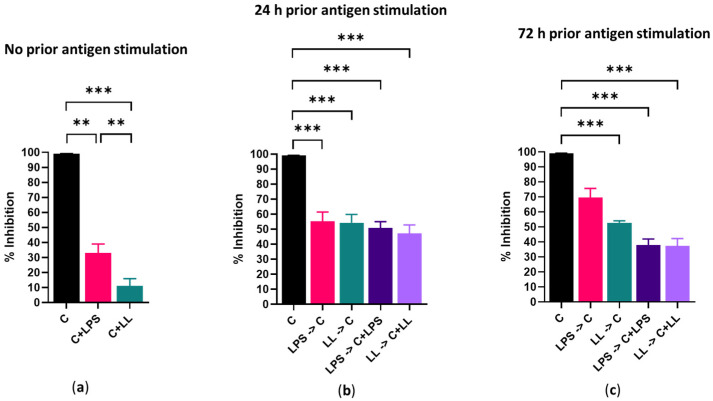
Effect of 150 µM/mL capsaicin on Cal 27 cell metabolism. Cal 27 cells were either with (**a**) no antigen pre-stimulation or (**b**) 24 h or (**c**) 72 h bacterial antigens pre-stimulation prior to the addition of inhibitory drugs. C = capsaicin, LPS = lipopolysaccharide, LL = LPS + LTA (lipoteichoic acid). Letter before the arrow represents pre-stimulation, e.g., LPS -> C is LPS pre-stimulation and then treatment with capsaicin (C). *n* = 9 biological replicates. Error bars represent standard error of the mean. ** *p* < 0.01, *** *p* < 0.001.

**Figure 5:**
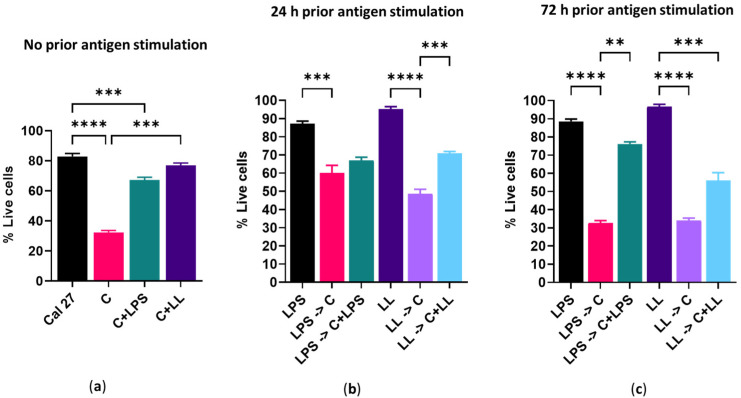
Effect of 150 µM/mL capsaicin on Cal 27 cell viability. Cal 27 cells were either with (**a**) no antigen pre-stimulation or (**b**) 24 h or (**c**) 72 h bacterial antigens pre-stimulation prior to the addition of inhibitory drugs. C = capsaicin, LPS = lipopolysaccharide, LL = LPS + LTA (lipoteichoic acid). Letter before the arrow represents pre-stimulation, e.g., LPS -> C is LPS pre-stimulation and then treatment with capsaicin (C). *n* = 9 biological replicates. Error bars represent standard error of the mean. ** *p* < 0.01, *** *p* < 0.001, **** *p* < 0.0001.

In the absence of prior bacterial antigenic stimulation, 24 h of capsaicin treatment reduced the metabolism of Cal 27 cells by 100% (Figure 4a) and killed 68% of Cal 27 cells (Figure 5a). The addition of bacterial antigens prior to capsaicin treatment significantly reduced metabolic inhibition by 30–50% (*p* ≤ 0.001) (Figure 4b,c) and improved cell viability (Figure 5b,c).

The addition of antigens at the same time as capsaicin even without prior antigenic stimulation significantly reduced drug efficacy as both cell death and metabolic inhibition were significantly reduced (*p* ≤ 0.001), and this effect was significantly greater with combined LPS + LTA than with LPS stimulation alone (*p* ≤ 0.01) (Figure 4a and Figure 5a). 

The addition of antigen prior to and concurrently with capsaicin treatment reduced the effect of the capsaicin treatment, resulted in significantly reduced Cal 27 cell metabolism inhibition (*p* ≤ 0.001) (Figure 4b,c) and significantly improved cell viability when compared with cells treated with capsaicin only (Figure 5b,c). For example, without prior bacterial antigenic stimulation, cell viability with capsaicin treatment alone was 32% (Figure 5a). However, with 24 h prior LPS + LTA stimulation, cell viability was increased to 49%, and when LPS + LTA was added with the capsaicin during treatment with or without prior antigen stimulation, cell viability was 71% (*p* ≤ 0.001) (Figure 5b). This suggests that the presence of antigen during treatment reduces drug efficacy, more so than the presence of antigen prior to the commencement of treatment.

### 2.4. Modulation of Capsaicin Induced Apoptosis by Bacterial Antigens

**Figure 6:**
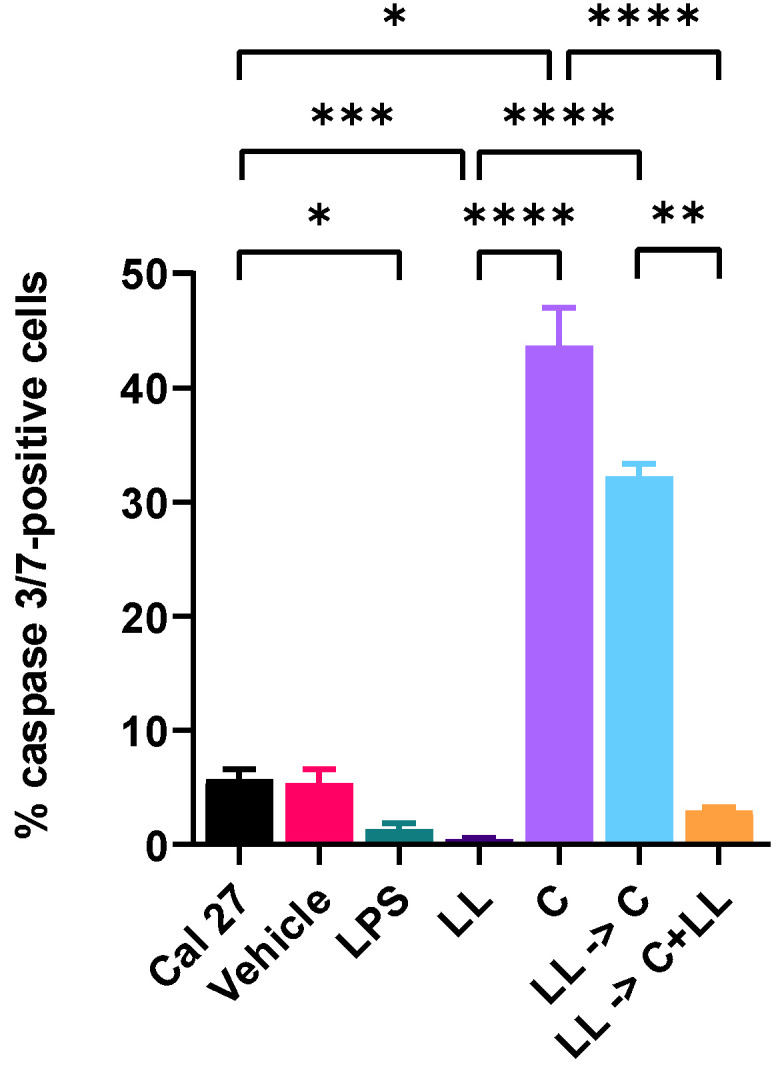
Percentage of apoptotic cells as determined by caspase 3/7-positivity. After Cal 27 cells were stimulated with LPS + LTA for 72 h, Cal 27 cells were treated with capsaicin for 24 h in the absence or presence of oral bacterial antigens. Vehicle (0.1% DMSO), LPS (5 µg/mL), LL = combined LPS (5 µg/mL) + LTA (5 µg/mL), C = capsaicin. Letter before the arrow represents pre-stimulation, e.g., LPS -> C is LPS pre-stimulation and then treatment with capsaicin. * *p* < 0.05, ** *p* < 0.01, *** *p* < 0.001, **** *p* ≤ 0.0001 are statistically significant at *n* = 9 biological replicates. Error bars represent standard error of the mean.

Capsaicin treatment of Cal 27 cells resulted in 43.6% ± 3.3% of the cells being apoptotic. However, capsaicin treatment following 72 h stimulation of Cal 27 cells with LPS + LTA reduced apoptosis to 32.2% ± 1.1% of cells. This was reduced even further to 2.98% ± 0.3% (*p* < 0.01) when capsaicin treatment happened in the presence of bacterial antigens. Treatment with capsaicin in the absence of bacterial antigens resulted in significantly more apoptotic cells than in the presence of bacterial antigens (*p* < 0.0001) (Figure 6).

### 2.5. Effect of Capsaicin Treatment on Gene and Protein Expression of Oral Cancer Cell Proliferation Factors

**Figure 7:**
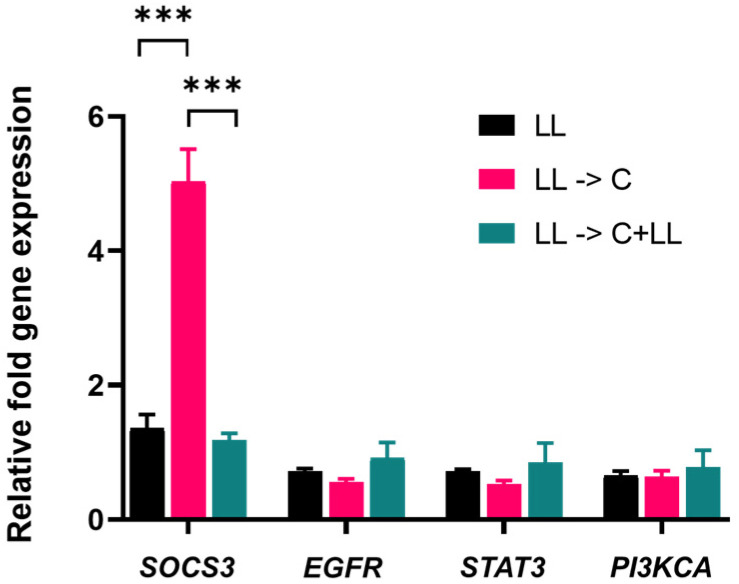
Expression of tumour suppressor and proliferation-related proteins by RT-qPCR analysis in Cal 27. Cal 27 cells were stimulated with LPS + LTA (LL) for 72 h, and then treated with capsaicin for 24 h in the absence (LL-> C) or presence (LL-> C + LL) of oral bacterial antigens. All the gene expressions are relative to Cal 27 cells without bacterial antigen stimulation and capsaicin treatment. C: capsaicin, LL = LPS + LTA. Letter before the arrow represents pre-stimulation, e.g., LL-> C is LL pre-stimulation and then treatment with capsaicin. *** *p* ≤ 0.001 is statistically significant at *n* = 9 biological replicates. Error bars represent standard error of the mean.

Real-time reverse transcription quantitative PCR (RT-qPCR) analysis of different tumour suppressor and proliferation-related proteins in Cal 27 are shown in Figure 7. After 72 h stimulation of Cal 27 cells with LPS + LTA, capsaicin treatment significantly increased gene expression of *SOCS3* of oral cancer cells (*p* ≤ 0.001). Interestingly, the *SOCS3* gene expression was reversed in the presence of bacterial antigens (*p* ≤ 0.001). There was no significant *EGFR*, *STAT3* and *PI3KCA* gene expression difference among the treatment groups. Western blot confirmed the results for EGFR, STAT3 and PI3K in protein expression, however, although *SOCS3* mRNA expression was significantly increased, there was no corresponding increase in SOCS3 protein expression (Supplementary Figure S1).

## 3. Discussion

In the absence of antigenic stimulation, 150 µM capsaicin reduced the metabolism of Cal 27 cells by 100% and killed 68% of the cells. However, the addition of bacterial antigens prior to drug treatment reduced the efficacy of capsaicin treatment. Simultaneous addition of bacterial antigens with drug treatment with and without prior bacterial antigen stimulation also significantly reduced capsaicin efficacy, resulting in decreased cell killing and decreased inhibition of metabolism. In general, the addition of both LPS and LTA resulted in greater inhibition of drug efficacy than the addition of LPS only. We believe the mechanism for this decreased efficacy is that bacterial antigens increase cancer cell proliferation. Other researchers using a co-culture system found that cancer drug efficacy was significantly changed due to the presence of bacteria [13].

The most challenging aspect of the project was to design a drug combination treatment that could mimic the real-world challenge of anti-cancer drugs in the presence of bacterial antigens that themselves mimic the presence of Gram-negative and Gram-positive bacteria in dental plaques containing oral biofilms. Oral biofilm formation is a continuous process. Even after proper extirpation/elimination of a bacterial plaque via surgical manoeuvre or antibiotic treatment, oral cancer patients frequently experience unhygienic oral conditions, leading to continuous formation of bacterial biofilm. The elimination of a bacterial plaque during treatment does not necessarily remove the existing bacterial antigen stimulatory effect on oral cancer cells. Therefore, combination drug treatment may be an appropriate approach to elucidate the confounding effects of bacterial antigens on chemotherapeutic drugs.

Capsaicin treatment significantly increased gene expression of *SOCS3* of oral cancer cells. However, the increased *SOCS3* gene expression was reversed in the presence of bacterial antigens during treatment. SOCS3, considered to be a tumour suppressor protein, primarily acts on the JAK/STAT pathway [14] and regulates proliferation and activation of T-helper cells [15]. Loss of *SOCS3* gene expression turns STAT3 from anti-apoptotic to pro-apoptotic [16]. Hyperactivation of EGFR mediates apoptosis via STAT3 [17], and EGFR hyperactivation may result in lowering of cell viability, independent of the PI3K/Akt pathway [18]. However, there was no significant difference between *EGFR* and *STAT3* gene and protein expression in capsaicin-treated pre-stimulated cells. Despite a significant increase in *SOCS3* mRNA expression, a corresponding increase in protein expression was not detected. The gene expression found in mRNA levels may not necessarily correspond to protein levels. 

Recent evidence supports the role of microbiota in cancer development and carcinogenesis, especially oral cancer [2,19] and colon cancer [20]. To the best of our knowledge, this is the first study to investigate the effect of bacterial antigens on oral cancer treatment, mimicking the real-world scenario. 

However, there are some limitations in this study. The limitations and possible future studies are discussed below.

First, this project was not carried out in a co-culture model setup (i.e., oral bacteria and oral cancer cells were cultured together) that incorporated immune cells. Bacterial antigens have been found to mostly target immune cells for cancer progression. The project found that bacterial antigens can stimulate oral cancer cells without the presence of immune cells. However, in the oral cavity, there are confounding factors such as immune cells and cytokines, and future studies using an immune cell–cancer cell co-culture model, or a tissue study on T-cell phenotypic changes, should be undertaken to elucidate the role of bacterial antigens and capsaicin more thoroughly.

Second, the project only used one oral cancer cell line (Cal 27), which is an aggressive cancer cell line and has been shown to respond to bacterial antigen stimulation more than SSC4, SSC9 and SSC25 cell lines [9]. The effect of bacteria antigens on cancer drug efficacy needs to be determined in additional cancer cell lines. Cancer tissues are heterogeneous in nature, and the effect of bacterial antigens and capsaicin based on one oral cancer cell line may not be generalised to other cancer cell lines.

Third, this project used LTA and LPS to simulate Gram-positive and Gram-negative bacterial infection, respectively. However, components of bacteria other than their bacterial cell wall antigens may also be responsible for the progression of oral squamous cell carcinomas. The project could not establish a temporal relationship in the effect of different bacterial antigens on the proliferation of the oral cancer cells. During the formation of a biofilm, Gram-positive bacteria are the initial colonisers. Gram-positive bacterial antigen may result in the upregulation or downregulation of various proliferating, apoptotic or anti apoptotic factors before Gram-negative bacterial antigens have any effect on oral cancer cells.

Fourth, the project has only investigated the effect of bacterial antigens on one anticancer agent—capsaicin. Cancer development and progression is a complex process, involves many pathways and there are many other cancer target and anticancer agents. Future studies should explore the effect of bacterial antigens on other anticancer agents.

## 4. Materials and Methods

**Figure 8:**
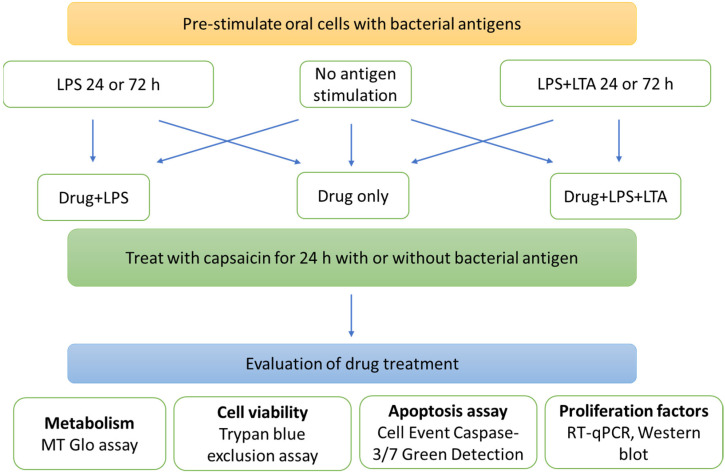
Experiment overview. Cal 27 and OKF6 cells were pre-stimulated with no bacterial antigen, LPS 5 µg/mL or combined LPS 5 µg/mL + LTA 5 µg/mL for 24 or 72 h. The media was removed and cells were treated with capsaicin, in the presence or absence of bacterial antigens for 24 h prior to harvesting and determination of the drug effect on proliferation, apoptosis and expression of proliferating factors.

An experiment overview is displayed in Figure 8. Details of all materials and reagents used in this study are listed in Supplementary Table S1, and details of oral cell lines used in the study are listed in Supplementary Table S2.

### 4.1. Cell Lines and Culture Conditions

Oral cancer cells Cal 27 (American Type Culture Collection CRL-2095, Manassas, VA, USA) were used for this study, as we previously showed that LPS and LTA stimulation resulted in greater proliferation in Cal 27 cells than in other oral cancer cell lines (SSC4, SSC9 and SS25) or normal oral cell lines (*p* ≤ 0.001) tested [9]. Cal 27 cells were cultured using Dulbecco’s Modified Eagle Medium (DMEM) supplemented with 10% foetal bovine serum and 1% penicillin/streptomycin, while OKF6 (normal oral cells) were cultured in keratinocyte serum-free medium (KSFM) plus growth factors, as previously described [9]. Cells were seeded at 5000 cells per well in a 96-well plate as previously described [9]. Each 75 cm^2^ flask of cells acted as a biological replicate, and each flask was obtained from three different cell passages. Experiments were repeated at different times. Throughout this report, *n* refers to the number of biological replicates.

### 4.2. Determine the Capsaicin Treatment Time and Concentration

Cal 27 cells were incubated with capsaicin (Sigma-Aldrich, St. Louis, MO, USA) at varying concentrations from 0 to 150 µM for 24 h, and cellular metabolism was determined by the MT Glo assay. The level of inhibition remained steady for up to 24 h (Figure 2b). In contrast, mitogens such as LPS or LTA require a longer time for activation of cells, and therefore, the capsaicin efficacy tests were conducted for 24 h.

### 4.3. Bacterial Antigens and Capsaicin Combination Tests

The clinical rationale of bacterial antigens’ stimulation and capsaicin treatment is explained in Figure 1. Bacterial antigens, LPS (from *Escherichia coli* O111:B4, Sigma Aldrich, St. Louis, MO, USA) and LTA (from Streptococcus pyogenes, Sigma Aldrich, St. Louis, MO, USA) were used at their previously found optimum concentration of 5 µg/mL [9], and capsaicin at 150 µM.

Cal 27 cells were plated in a 96 well flat-bottomed plate at 5000 cells/well and stimulated with bacterial antigens LPS/LPS + LTA for 0, 24 and 72 h prior to treatment with capsaicin in the presence or absence of additional antigenic stimulation for 24 h. Then, cellular metabolism and viability were determined (Figure 8). Cal 27 cells without capsaicin treatment were used as control cells for each condition.

### 4.4. Inhibition Effect of Capsaicin Treatment on Oral Cancer Cell Metabolism

Cell metabolism was determined using the real-time Glo MT cell viability assay (Promega) according to the manufacturer’s instructions.

The percentage of inhibition of metabolism of capsaicin treatment was calculated as:

(1) $$\%\;\mathrm{Inhibition}\;=\;\left[\frac{\mathrm{Luminescence}\;\mathrm{of}\;\mathrm{the}\;\mathrm{control}\;\mathrm{cells}-\;\mathrm{Luminescence}\;\mathrm{of}\;\mathrm{the}\;\mathrm{treated}\;\mathrm{cells}}{\mathrm{Luminescence}\;\mathrm{of}\;\mathrm{the}\;\mathrm{control}\;\mathrm{cells}}\right]\times 100$$

### 4.5. Cell Viability Assay

Cell viability was determined by the Trypan blue exclusion assay. Cells were detached by the addition of 40 µL of 0.25% trypsin EDTA solution (Sigma, St. Louis, MO, USA), mixed in an equal volume of 0.4% Trypan blue (Invitrogen, Waltham, MA, USA), and live and dead cells were counted.

### 4.6. Apoptotic Assay

Caspase 3/7-positive cells are a marker of apoptosis. Cell Event Caspase-3/7 Green Detection Reagent (Invitrogen, Waltham, MA, USA) was used for live cell imaging and to estimate the total number of apoptotic cells, and 5000 cells (Cal 27) per 96-well plate (transparent, flat-bottomed) were plated and subjected to different treatments. After the incubation period for each treatment, the medium was carefully removed, and cells were washed once with 1× PBS, then 100 µL of 7.2 µM Cell Event Caspase-3/7 Green Detection Reagent in 1× PBS + 5% FBS was added to each well of the 96-well plate and incubated at 37 °C/5% CO_2_ for 30 min. The cells were imaged using the FITC filter sets, as the maximum excitation/emission for the Cell Event Caspase-3/7 Green Detection Reagent is 502/530 nm. Caspase3/7 apoptotic analysis was carried out using ImageJ version 1.52a software (National Institutes of Health, Bethesda, MD, USA, http://imagej.nih.gov/ij (accessed on 10 August 2021)).

### 4.7. Reverse Transcription Quantitative PCR for Proliferation Factors

Reverse transcription quantitative PCR was conducted for proliferation factors *SOCS3*, *EGFR*, *STAT3*, *GAPDH* and *PI3KCA*. RNA extraction and RT-qPCR and analysis were performed as described previously [9]. Briefly, total RNA was extracted with the Invitrogen Trizol Plus RNA purification kit. SSIV VILO Master Mix W/EzDNase (Invitrogen, Waltham, MA, USA) was used to reverse transcribe the RNA and qPCR conducted using Power-up SYBR Master Mix (Invitrogen, Waltham, MA, USA). Primer pair sequences are listed in Supplementary Table S3. The house-keeping gene GAPDH (internal control) and the untreated Cal 27 cells were used to normalise the gene expression fold change in the treatment groups using the comparative Ct (ΔΔCt) method.

### 4.8. Western Blot

Ten μg of total protein from each bacterial antigen treatment condition was used for Western blotting. Primary antibodies were obtained from R&D Systems and used at the following concentrations: 0.1 μg/mL human/mouse SOCS3 antibody, 1 μg/mL human PI 3-kinase p110β antibody, 0.1 μg/mL STAT3 Mouse anti-human, mouse, rat, 1 μg/mL human EGFR antibody, 1:1000 human GAPDH. Blots were probed with the respective secondary antibodies from R&D Systems at the following concentrations: 1:1500 anti-mouse IgG HRP conjugate, 1:1500 anti-goat IgG HRP conjugate, 1:1500 anti-rabbit IgG HRP conjugate. High-resolution images were acquired using the Chemidoc MP Imaging system and processed using Image Lab version 6.0 (Bio-Rad Laboratories, Hercules, CA, USA). The band densities of the house-keeping gene GAPDH (internal control) and the untreated Cal 27 cells were used to normalise the protein expression in the treatments [9].

### 4.9. Statistical Analysis

All statistical analyses were performed using GraphPad Prism version 9 (GraphPad Software, San Diego, CA, USA). The data were tested for normality of distribution using the D’Agostino and Pearson omnibus normality test. One-way or two-way analysis of variance (ANOVA) Kruskal–Wallis test multiple comparisons on mean ranks were performed in the oral cell proliferation and suppression, metabolism, viability and gene expression analyses. The probability threshold of *p* ≤ 0.05 was considered statistically significant.

## 5. Conclusions

This study demonstrated that bacterial antigens play a confounding role during the action of capsaicin on Cal 27 oral cancer cell proliferation. Capsaicin reduces bacterial antigen-induced oral cancer cell proliferation. The presence of bacterial antigens lowered the inhibition percentage of capsaicin, and combined LPS + LTA acted synergistically to lower the effect of capsaicin on Cal 27 oral cancer cells.

These findings may benefit other cancer researchers considering the presence of bacterial antigens when developing novel pharmacological strategies for cancer, not only oral cancer, head and neck cancer, but also colorectal cancer, where commensal bacteria may affect chemotherapeutic drugs. The importance of eliminating the bacterial antigens is also highlighted, such as thoroughly cleaning the dental plaque and maintaining oral hygiene prior to and during oral cancer chemotherapy.

## Data Availability

The date presented in this study are available in Supplementary date file.

## Supplementary Materials

The following are available online at www.mdpi.com/article/10.3390/ijms22168686/s1.
