# Supplementary material for: Bacterial Antigens Reduced the Inhibition Effect of Capsaicin on Cal 27 Oral Cancer Cell Proliferation
Source: Int J Mol Sci. 2021 Aug 12;22(16):8686. doi: 10.3390/ijms22168686 (PMC8395464; doi:10.3390/ijms22168686)
Supplement: Supplementary file 1 [file ijms-22-08686-s001.zip › Supplementary Material.pdf]

Supplementary Material

Bacterial Antigens Reduced the Inhibition Effect of Capsaicin on Cal 27 Oral Cancer Cell Proliferation

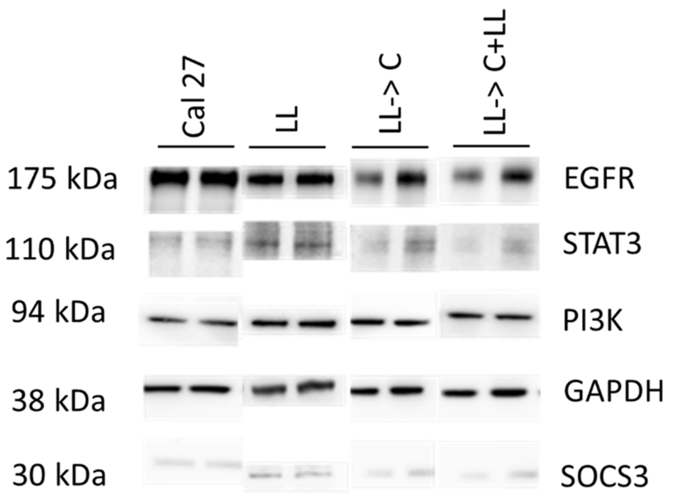

Figure S1. Western blot image of different tumour suppressor and proliferation related proteins in Cal 27. Cal 27 cells were stimulated with LPS + LTA (LL) for 72 h, and then treated with capsaicin for 24 h in the absence (LL -> C) or presence (LL -> C+LL) of oral bacterial antigens. All the gene expressions are relative to Cal 27 cells without bacterial antigen stimulation and capsaicin treatment. C = capsaicin, LL = LPS + LTA. Letter before arrow represents pre-stimulation e.g., LL -> C is LL pre-stimulation and then treatment with capsaicin.

Table S1. Detail of materials and reagents used in the study.

**Reagent/Kit**	**Manufacturer**	**LOT**	**Catalogue No.**	**Description**
		**Growth Medium and Buffers for the Cell Culture**		
Keratinocyte-Serum Free Medium (K-SFM)	Life Technologies	-	1700504	Contains 2.5 μg human recombinant epithelial growth factor (EGF) (0.035 μg/μL) and 25 mg bovine pituitary extract (15.1 mg/mL)
Foetal bovine serum (FBS)	Life Technologies	-	10099141	Endotoxin level: ≤ 5 EU/mL. Haemoglobin level: ≤ 30 mg/dL (levels routinely ≤ 25 mg/dL).
1X phosphate buffered saline (PBS)	Gibco	-	20012-027	1.5 mM KH_2PO_4; 155.2 mM Cl; 2.7 mM $Na_2HPO_4.7H_2O$ (pH 7.2)
Trypsin-EDTA	Sigma Life Sciences	SLBZ7364	T4049	0.25%, bioreagent 2.5 gm porcine trypsin and 0.2 gm EDTA; 4 Na per litre of Hank's balanced salt solution with phenol red
Dulbeccos Modified Eagles Medium (DMEM)	Gibco	-	11960-044	4500 mg/L glucose, sodium pyruvate and sodium bicarbonate
Penicillin–streptomycin (P/S) solution	Life Technologies	2068825	15140-148	10,000 units/mL penicillin and 10,000 μg/mL streptomycin
		**Drugs and Bacterial Antigens**		
LPS 25 mg	Sigma-Aldrich	-	L2630	Lipopolysaccharides from *Escherichia coli* O111:B4, water soluble
LTA 5 mg	Sigma-Aldrich	049M4155V	L3140	Lipoteichoic acid from *Streptococcus pyogenes*, water soluble
Capsaicin 250 mg	Sigma-Aldrich	MKBV4243V	360376	8-Methyl-N-vanillyl-trans-6-nonenamide, polar compound soluble in organic solvent
		**Proliferation Assay**		
RealTime-Glo MT Cell Viability Assay	Promega	0000383765	G9712	10 × 100 reactions
MT Cell Viability Substrate, 1000X	Promega	0000362418	G971A	10 μL solution supplied in opaque tubes
NanoLuc Enzyme, 1000X	Promega	0000362418	E499A	0.4 mg/mL

Trypan blue stain 0.4%	Invitrogen	2117644	T10282	Trypan blue 0.4% to identify dead cells via colorimetric detection; supplied as 21 mL vials
Crystal violet acetate	Sigma Aldrich	69H3634	C5042	9(amino-5-imino-5H-benzo[a] phenoxyazine acetate salt suppled as powder
Methanol	Chem-Supply	UN1230	MA004-2.5L-P	Analytical reagent
Cell Countess cell-counting chamber slides	Invitrogen	I31A9 Q422	C10283	For live cell and dead cell percentage via calorimetric detection
**Apoptotic Assay**				
CellEvent Caspase-3/7 Green Detection Reagent 25 µl	Invitrogen	2119122	C10723	2.0 mM solution in DMSO
**Determination of Proliferation Related Gene Expression**				
Pure Link RNA mini kit	Invitrogen	2137737	12183018A	Total RNA extraction
Trizol reagent	Invitrogen	260702	15596026	Total RNA extraction
Ethyl alcohol	Sigma Aldrich	SHBH7551	E7023-1L	CH_3CH_2OH; pure
SSIV VILO Master mix W/ EzDNASE	Invitrogen	00831746	11766050	DNA digestion and Reverse transcription PCR
PowerUp SYBR Master Mix, 5 ml	Invitrogen	00799448	A25742	Real-time PCR Master Mix
Chloroform	BDH AnalaR	19073	VWRC22711.260	$CHCl_3$, contains 1% *v*/*v* of ethanol as preservative
MicroAmp Fast plate	Applied Biosciences, Life Biosystems	-	4346907	96-well reaction plate (0.1 mL)
Optical adhesive cover	Applied Biosystems, Life Technologies	-	4360954	qPCR-compatible optical adhesive covers
**Protein Estimation**				
Pierce BCA Protein Assay kit	Thermo Fisher Scientific	UD2969	23225	Two-component, high-precision, detergent-compatible protein assay for determination of protein concentration
**Western Blot**				
Human/Mouse SOCS-3 MAb (Clone 516919), 25 µg	R&D Systems	CCDL0219051	MAB5696	Purified mouse monoclonal IgG
Human/Mouse/Rat STAT3 MAb (Clone 232209), 25 µg	R&D Systems	JXW041912A	MAB1799	Purified mouse monoclonal IgG
Human PI 3-Kinase p110 beta MAb (Clone 269020), 25 µg	R&D Systems	VCK042001A	MAB2686	Purified mouse monoclonal IgG
Anti-G3PDH/GAPDH (T0893), 20 µL	R&D Systems	20481	2275-PC-020	Polyclonal rabbit antibody
Human EGF R/ErbB1 Polyclonal Ab, 25 µg	R&D Systems	AUC1118111	AF231	Affinity purified goat IgG
Novex Sharp pre-stained protein standard	Life Technologies	2115579	LC5800	consists of 12 pre-stained protein bands in molecular weight range 3.5–260 kDa
Clarity Western ECL Substrate	Bio-Rad	102031366 102031363	1705060	Supplied in two parts: peroxide solution and luminol/enhancer solution
Ponceau S solution	Sigma-Aldrich	SLCB3855	P7170-1L	Bioreagent 0.1% (*w*/*v*) supplied in 5% acetic acid: $C_{22}H_{12}N_4Na_4O_{13}S_4$
Anti-goat IgG HRP conjugate	R&D Systems	XGD10161011	HAF009	Secondary antibody specific to primary antibody source
Anti-rabbit IgG HRP conjugate	R&D Systems	FIN1819021	HAF008	Secondary antibody specific to primary antibody source
Anti-mouse IgG HRP conjugate	R&D Systems	WVA00919011	HAF018	Secondary antibody specific to primary antibody source
NuPAGE LDS sample buffer (4X)	Invitrogen	1981103	NP0007	Used to prepare protein samples for denaturing gel electrophoresis with Bis-Tris or Tris-Acetate gels. It contains lithium dodecyl sulfate, pH 8.4; contains Coomassie G250 and Phenol Red
NuPAGE 10% bis-tris gel	Invitrogen	19050170	NP0302BOX	1 mm × 12 well
NuPAGE 10% bis-tris gel	Invitrogen	19071070	NP0301BOX	1 mm × 10 well
20X NuPAGE MOPS SDS Running Buffer	Invitrogen	-	NP0001	500 mL contains 50 mM MOPS, 50 mM Tris Base, 0.1% SDS, 1 mM EDTA, pH 7.7

Table S2. Details of oral cell lines.

**Cell Line**	**OKF6**	**CAL 27**
Source	Cellosaurus	ATCC
Specimen site	Floor of mouth; normal oral keratinocytes	Tongue squamous cell carcinoma
Type	Immortalised normal oral cells	Primary
Treatment	No	No
Sex	Male	Male
Age (years)	57	56
Culture media	K-SFM media + growth factors	DMEM + 10% FBS + P/S
Freezing media	50% FBS + 10% DMSO + DMEM	50% FBS + 10% DMSO + DMEM

Table S3. Primers used in RT-qPCR for proliferation factors.

**Gene Name**	**Primer Pair Sequences (5′ to 3′)**
SOCS3	GCGCGAAGGCTCCTTTGTG
	GGGGGGCTGGTCCCGAATC
STAT3	GGACATCAGCGGTAAGACCC
	CTCTGGCCGACAATACTTTC
EGFR	AGCTACGGGGTGACTGTTTG
	GAACTTTGGGCGACTATCTG
GAPDH	GACAGTCAGCCGCATCTTCT
	ACCAAATCCGTTGACTCCGA
PI3KCA	TGGGGATGATTTACGGCAAG
	TCCCACACAGTCACCGATTGA
